# The genetic structure of Norway

**DOI:** 10.1038/s41431-021-00899-6

**Published:** 2021-05-17

**Authors:** Morten Mattingsdal, S. Sunna Ebenesersdóttir, Kristjan H. S. Moore, Ole A. Andreassen, Thomas F. Hansen, Thomas Werge, Ingrid Kockum, Tomas Olsson, Lars Alfredsson, Agnar Helgason, Kári Stefánsson, Eivind Hovig

**Affiliations:** 1 Centre for Coastal Research, Department of Natural Sciences, University of Agder, Kristiansand, Norway; 2 Center for Bioinformatics, Department of Informatics, University of Oslo, Oslo, Norway; 3 deCODE Genetics/AMGEN, Inc., Reykjavik, Iceland; 4 Department of Anthropology, University of Iceland, Reykjavik, Iceland; 5 NORMENT, Division of Mental Health and Addiction, Oslo University Hospital, Oslo, Norway; 6 Institute of Clinical Medicine, University of Oslo, Oslo, Norway; 7 Institute of Biological Psychiatry, Copenhagen Mental Health Services, Copenhagen, Denmark; 8 Danish Headache Center, Department of Neurology, Copenhagen University hospital, Glostrup, Denmark; 9 Department of Clinical Medicine, University of Copenhagen, Copenhagen, Denmark; 10 The Lundbeck Foundation Initiative for Integrative Psychiatric Research, iPSYCH, Copenhagen, Denmark; 11 Department of Clinical Neuroscience, Center for Molecular Medicine, Neuroimmunology Unit, Karolinska Institutet, Stockholm, Sweden; 12 Institute of Environmental Medicine, Karolinska Institutet, Stockholm, Sweden; 13 Faculty of Medicine, University of Iceland, Reykjavik, Iceland; 14 Department of Tumor Biology, Institute for Cancer Research, Oslo University Hospital, Oslo, Norway

**Keywords:** Genetic variation, Genetic markers

## Abstract

The aim of the present study was to describe the genetic structure of the Norwegian population using genotypes from 6369 unrelated individuals with detailed information about places of residence. Using standard single marker- and haplotype-based approaches, we report evidence of two regions with distinctive patterns of genetic variation, one in the far northeast, and another in the south of Norway, as indicated by fixation indices, haplotype sharing, homozygosity, and effective population size. We detect and quantify a component of Uralic Sami ancestry that is enriched in the North. On a finer scale, we find that rates of migration have been affected by topography like mountain ridges. In the broader Scandinavian context, we detect elevated relatedness between the mid- and northern border areas towards Sweden. The main finding of this study is that despite Norway’s long maritime history and as a former Danish territory, the region closest to mainland Europe in the south appears to have been an isolated region in Norway, highlighting the open sea as a barrier to gene flow into Norway.

## Introduction

Population sub-structures can give rise to false-positive associations in association studies of genetic variants [1], can reveal historical patterns of population movements [2, 3], and estimates of ancestry have potential in informing genealogy and forensic genetics [4]. Norway with its natural features, such as the sea and mountain ridges, tends to limit gene flow between groups of individuals [5], resulting in reproductive isolation and divergence in allele frequencies over time. This divergence may be especially pronounced in smaller populations, due to greater genetic drift. Among the populations in Northern Europe, geographically structured differences are primarily due to isolation by distance, but may also result from founding effects and subsequent isolation [6, 7]. Further, isolation and reduction of gene flow within a geographical area can also manifest an increase in recessive Mendelian disorders [8, 9] and founder variants. Indeed, geographically clustered and expanding BRCA1 founder variants have been previously reported for Norway [10, 11].

Norway is one of the most sparsely populated countries in Europe, but little is known about its main genetic structure. Its relatively large landmass has the longest coastline in Europe but has a population of only ~5 million, which includes one of the few indigenous peoples of Europe, the Sami. With unfavorable climatic conditions, combined with the third least arable land in Europe, Norway has provided its people with limited agricultural opportunities. Historically, farms were fragmented through inheritance to ever smaller units, ultimately resulting in unsustainable population growth, especially during the 19th century. Combined with poverty, this motivated the mass emigration of a substantial fraction (1/3) of the population to the Americas during the 19th century, a fraction only surpassed by Ireland [12]. Despite recent urbanization, leading to one-third of the population residing in cities with >100,000 inhabitants, Norway remains characterized by rural communities and small coastal cities. The diversity in dialects across the country suggests limited gene flow in the past [13].

As might be expected, genetic studies show that contemporary Norwegians are most closely related to the neighboring populations of Sweden and Denmark [14, 15]. Genetic studies of the human populations of Denmark, Sweden, Finland, and Iceland have revealed some intriguing results, highlighting the impact geography has on human genetic variation and admixture, including minimal structure in the Danish population [15], a north-south gradient in Sweden [16] and founder effects and genetic drift in Finland [6, 17] and Iceland [14, 18, 19].

Here, we describe the geographical structure of the Norwegian gene pool in detail, based on microarray genotypes from 6369 unrelated individuals from a biobank of self-reported overrepresentation of cancer in their families, who were assigned geographical coordinates based on postal codes. As the mean age of these individuals is approximately 64 years, our analysis provides an overview of stratification in the Norwegian gene pool prior to recent episodes of immigration [20, 21].

## Materials and methods

### Samples

The dataset was derived from a biobank of approximately 18,000 EDTA-contained blood samples collected over a period of 25 years, as a patient self-referral initiative for overrepresentation of cancer in families, with both clinical and research intent. It includes information about family structure and place of residence as postcodes, which were converted into longitude and latitude coordinates [22]. The biobank consists of families, as well as unrelated individuals, with partial pedigree information covering more than 50,000 individuals [10, 11]. Its clinical aim was to provide benefit to patients from the established follow-up examinations aiming at early diagnosis and treatment. All participants provided separate written informed consent to the current research, and the study was approved by the regional ethical review board (REK sør-øst C: 2015/2382).

### Genotypes and sample quality control

DNA was extracted and genotyped at deCODE genetics using the Illumina OmniExpress 24 v 1.1 chip, containing assays for 713,014 SNPs. Data analyses were performed both on the “Services for sensitive data” (TSD) platform at the University of Oslo and at deCODE genetics. The genotyped samples were subjected to quality control and processing in the following order (Supplementary Table S1), using PLINK (v1.90b3) [23]. First, we removed SNPs on sex chromosomes. Then autosomal SNPs with a missing rate >2% were removed, followed by removal of SNPs with a minor allele frequency (MAF) < 2%. Next, samples with more than 2% missing data were excluded, along with those without a postal code. This resulted in 583,183 autosomal SNPs typed in 14,429 individuals remaining. Finally, we identified all pairwise relationships between individuals using the “–related–degree 3” parameter in KING (v 1.2.3) [24], and discarded individuals related up to the third degree, keeping the oldest individual in each lineage. This resulted in a dataset of 6545 individuals with no close relations (kinship coefficient <0.044) and a mean age of 64 years. There was a predominance of females (81%) as the samples were collected through self-referrals for breast cancer.

As our focus is on population events that occurred prior to the second half of the 20^th^ century, we performed analyses to exclude individuals from our sample who derive from recent migration from distant populations. We assessed the extent of European (CEU), East-Asian (CHB), and African (YRI) ancestry in our Norwegian sample using ADMIXTURE (v 1.3.0) [25]. After examining the resulting distributions, we set the maximum threshold for African ancestry to 5%, leading to an exclusion of 65 individuals. The extent of East-Asian ancestry in our dataset was more pronounced (*n* = 141 > 5%). As many of these samples were found to be from the northernmost county of Finnmark, particularly from the Sami town of Kautokeino, we decided to set the Asian ancestry cutoff threshold >35% (excluding 29 samples), in order to retain individuals of presumed Sami ancestry. To determine if these indeed were of Sami ancestry, we merged our dataset with a public dataset with genotypes from individuals from a range of countries including one known Sami sample [26], and conducted a PCA. In total, we excluded 94 samples from further analysis that exceeded the thresholds of African (>5%) and East Asian ancestry (>35%). To verify that Asian ancestry in putatively Sami individuals was explained by Uralic-associated Siberian ancestry [27, 28] rather than recent ancestors from East Asia, we used the Human Origins dataset [26] and the R package *admixtools* (github.com/uqrmaie1/admixtools, retrieved 2021-02-01) to calculate *f*_*4*_ (Mbuti, *putative Sami individual*; Han Chinese, Nganasan) with *blgsize* = 500,000.

### Sample density

The samples in this study were distributed over most of Norway, with an over-representation of the south-eastern region that houses half the population, and an underrepresentation from the counties of Sogn og Fjordane and Finnmark (Table 1). For most analyses, we assigned individuals to one of the 19 counties of Norway based on postcodes and applied a restriction of a maximum of 200 random samples per county.

**Table 1:** Summary statistics per county.

County	Abb	*N*	N*	Median sum of ROH	Mean sum of IBD	Ne	Pop per km^2^	Pop	Ne/pop
Østfold	OF	388	200	5.5	5.7	396,000	56	221,386	1.79
Akershus	AK	1132	200	5	5.2	919,000	70	324,390	2.83
Oslo	OS	913	200	4.9	4.7	579,000	1127	481,548	1.20
Hedmark	HE	325	200	8	8.4	93,600	6	179,204	0.52
Oppland	OP	294	200	7.5	8.1	89,100	7	172,479	0.52
Buskerud	BU	388	200	5.6	7	204,000	14	198,852	1.03
Vestfold	VE	417	200	6	6.1	115,000	81	175,402	0.66
Telemark	TE	240	200	6.7	9.4	91,400	11	156,778	0.58
Aust-Agder	AA	152	152	8.2	10.2	118,000	9	80,839	1.46
Vest-Agder	VA	252	200	12	13.5	44,100	18	124,171	0.36
Rogaland	RO	225	200	8.4	14.2	27,600	31	268,682	0.10
Hordaland	HO	52	52	8.1	7.1	55,500	25	260,492	0.21
Sogn og Fjordane	SF	22	22	10.5	14.8	12,000	5	100,933	0.12
Møre og Romsdal	MR	187	187	7.8	9.6	270,000	15	223,709	1.21
Sør-Trøndelag	ST	1011	200	6.7	8.7	187,000	13	234,022	0.80
Nord-Trøndelag	NT	187	187	8.3	9.2	116,000	5	117,998	0.98
Nordland	NO	100	100	6.6	8	57,400	6	240,951	0.24
Troms	TR	54	54	8.8	11.5	25,600	5	136,805	0.19
Finnmark	FI	30	30	27	52.2	2600	2	39,757	0.07
All		6369	2984	6.8	–	–	12	3,888,305	–

*N* = the number of samples passing quality control. N* = the final number of random samples per county included in the final analysis, with max 200. Mean ROH = mean sum of Runs-of-Homozygosity in cM. Mean IBD = Mean within-county IBD sharing in cM. Ne = estimate of effective population size at *g* = 5 ago. Pop. size and pop. per km^2^ = census population size in 1970.

### Scandinavian dataset

The Norwegian dataset was merged with extended versions of the Danish and Swedish reference samples used in [14], genotyped on the same genotyping platform. SNPs passing quality control and filtering criteria in the Norwegian dataset were extracted from the Danish and Swedish datasets, expanding the dataset with 1853 Danish and 7966 Swedish samples.

### Principal component analysis and genetic distances

Linkage-disequilibrium (LD) was reduced by the use of a sliding window of 200 SNPs, stepping 25 SNPs and removing SNPs with r2 > 0.2 (PLINK: “–indep-pairwise 200 25 0.2”). After LD-pruning, we also excluded any SNPs present in any of the 24 regions with high LD [29, 30], which was subjected to principal component analysis (PCA) as implemented in the eigensoft v6.0.1 [7] function of smartPCA. The pairwise *F*_ST_ was calculated without automatic removal of outliers [31] and clustered using hierarchical clustering of the squared dissimilarities (ward.D2) and presented in a phylogram.

### Shared haplotypes and homozygosity

Missing data in the combined Scandinavian dataset were imputed without using a reference panel and phased using beagle v.5 [32]. Shared haplotypes, also known as identity-by-descent (IBD) segments, were detected for autosomal chromosomes using RefineIBD [33], using default settings (minimum length: 1.5 cM, lod > 3 in windows of 40 cM). We increased the minimum size of IBD to 3 cM in order to reduce the false discovery rate [33] [20303063] and summed pairwise IBD sharing between all possible pairs of individuals. Pairwise county-level ancestry was determined as the mean of the sum of IBD sharing between individuals residing in the counties in question. County information was available for Norway and Sweden, while Denmark was treated as one geographical unit.

The length of homozygous segments (cM) in each individual were summed to provide a measure of genomic inbreeding, the distribution of which was assessed by county (maximum *N* samples per county = 200, total *N* = 2984). To create a smoothed contour map of Norway, we combined the sum of homozygous content per individual with latitude and longitude in spatial regression as within the Krig function in the R package “fields” [2, 34].

### Historical effective population sizes

Temporal changes in effective population sizes can be estimated by the length and distributions of shared haplotypes (IBD) [35]. The effective size (*N*_e_) of a population can be assessed from the pattern of genetic variability in its gene pool and is affected by rates of migration and growth [36, 37]. Here, we implemented IBDne [35], for each county using IBD segments called by the RefineIBD algorithm [32, 38], assuming a generation time of 30 years [39]. IBDne was run with a minimum segment length of 3 cM. The remaining default parameters include minregion = 50 cM, trim cM 0,2, filtersample = true, npairs = data dependent, nboots = 80, gmax = 200, and seed = −99999.

### Estimation of migration rates and directed gene flow

Effective migration rates in Norway were estimated using EEMS [40], using the LD-pruned dataset. A spatial outline of Norway was constructed by representing it as a concave hull using the R package “concaveman”, and the resulting polygon was used as a border descriptor. A dissimilarity matrix using the bundled script “bed2diff” was constructed. The algorithm assigns individuals to the nearest deme, and by using a stepping-stone model, migration rates are estimated between demes. We used the default number of iterations of MCMC iterations = 2,000,000, burn-in iterations = 1,000,000, and a thinning interval of 9999, varying the deme sizes as 200, 500, and 800.

## Results

### Population structure in Norway

**Fig. 1:**
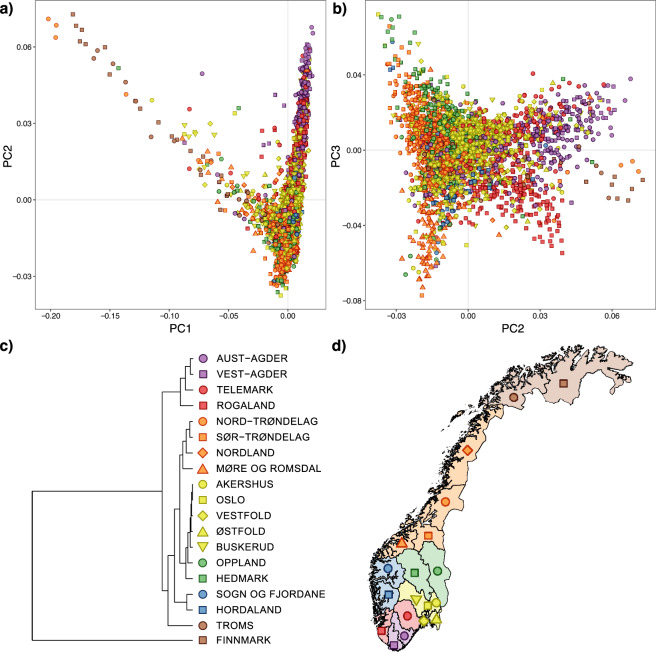
The genetic structure of Norway demonstrated by PCA and Fst values. **a**, **b** PCA plots of LD pruned SNPs (102,023) color-coded by county. PC1 captures the Sami component and PC2 a southern component of distinctive drift. **c** Hierarchical clustering of Reich’s *F*_st_ values, using squared dissimilarities (ward.D2) presented as a phylogram. **d** Color-coded map of the counties in Norway.

We performed a PCA to detect fine-scale population structure using LD-filtered SNPs (*n* = 102,023) (Supplementary Table S1). First, we color-coded the samples in the PCA (Fig. 1). The first component (PC1) captures the Uralic-associated admixture (Supplementary Fig. S1a), and variation in the second component (PC2) reflects differentiation in southern Norway. In order to mitigate the sample bias between the Norwegian sample and the public data resulting in the exaggeration of the Norwegian pattern, we also performed a PCA with a maximum of 20 individuals per county in Norway (Fig. S1b). This also demonstrates that the observed pattern of the genetic distance of Finnmark is not an artifact of undersampling, although the pattern may not be fully representative of the population. The geographical distribution of Uralic associated ancestry was quantified for each county using the results from admixture (Supplementary Fig. S2). Potential sources of Uralic ancestry include the indigenous Sami and later immigrating Finnish minorities. Using the *f*_*4*_ test (Mbuti, *X*; Han Chinese, Nganasan), we found that none of the 89 individuals *X* assigned >5% East Asian ancestry in ADMIXTURE showed significantly (±3 standard errors) more affinity to Han than to Nganasan, supporting the inference that they had Uralic-associated ancestry (Supplementary Fig. S10).

We also found evidence that the third (PC3) component captures meaningful geographical information (Fig. 1a, b). We assessed the relationships between PCs and geography (latitude and longitude) using a Pearson’s product-moment correlation coefficient test. PC1 showed significant (*p* < 2e−16) correlations with latitude (*r* = 0.42) and longitude (*r* = 0.44), as did PC2 (*p* < 2e−16; latitude *r* = −0.32, longitude *r* = −0.16). To further examine the correlation with geography, we color-coded the samples based on county and inspected the sample distribution in a PCA plot (Fig. 1a, b). The five postcodes with the largest and smallest mean scores in PC1 (*N* individuals >1) were: Kautokeino, Nesseby, Nordreisa, Røyrvik, and Alta in the northeast and Hægebostad, Hå, Eigersund, Birkenes, and Seljord in the South. A table of the municipality with mean PC1–10 values is available (10.6084/m9.figshare.11235803.v1).

To put the Norwegian population in a Scandinavian context, we conducted a PCA of the combined Scandinavian dataset. Here, the divergence of South Norway is apparent (Supplementary Fig. S3). In the first two PCs, there are three dimensions of divergence: Uralic-related ancestry, the Norwegian south, and the Swedish north.

### Genetic distances between Norwegian counties

Hierarchical clustering of pairwise *F*_ST_ distances between counties revealed a similar pattern as the PCA, with the largest divergence in Finnmark in the north, followed by the southern counties of Rogaland, Agder, and Telemark (Fig. 1c). We note that the counties Møre og Romsdal, Trøndelag, and Nordland group together, and that the counties by the Oslofjord area also form a cluster. The average pairwise *F*_ST_ between Norwegian counties was 0.0012 (max: 0.0073). For comparison, the mean pairwise *F*_ST_ values for regional differentiation in surrounding countries are: 0.0024 in Finland (max: 0.006), 0.0002 in Denmark, 0.0012 in Sweden (max: 0.0025), and 0.0007 in Great Britain (max: 0.003) [3, 15–17] (all *F*_ST_ values are derived from the same software (EIGENSOFT), except for the Danish study (PLINK)). Clearly, Finland stands out in this context, and Norway is comparable with Sweden in terms of inter-county differentiation. However, Norway has the largest extent of differentiation within a nation, with Rogaland vs. Finnmark, *F*_ST_ = 0.0073, which is also the most spatially distant (~1250 km) pairwise comparison in Scandinavia (we note that the Swedish study excluded samples with Uralic related ancestry) [16]. The aforementioned studies have used different genotyping platforms, and thus the derived Fst values have some limitations in directly comparing the values, but the main pattern of inter-county differentiation within the respective countries is likely to persist.

### Kinship and inbreeding in Norwegian counties

**Fig. 2:**
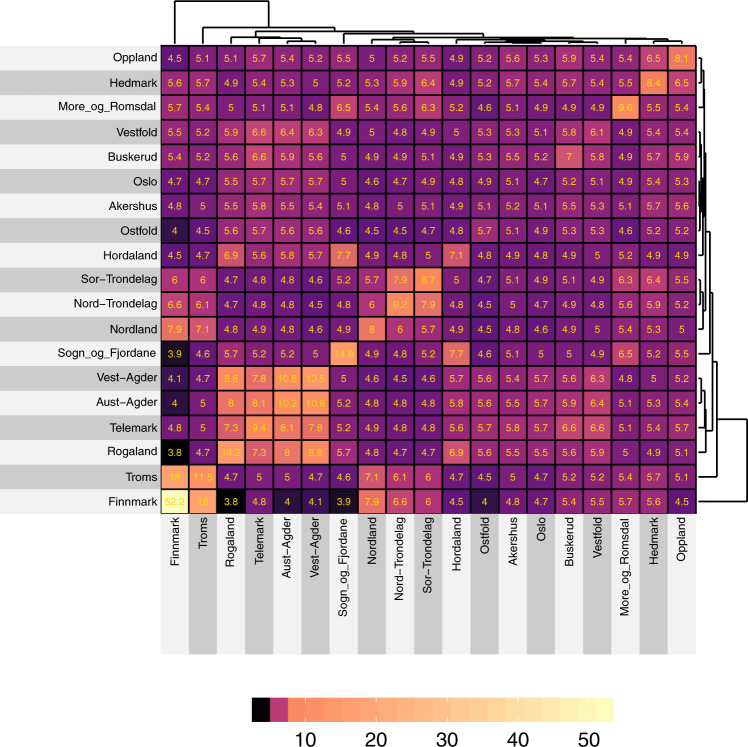
Visual representation and hierarchical clustering of the mean cumulative sum of haplotype sharing (IBD > 3 cM) within and between counties in Norway, in centiMorgans (cM). Overall, there is an increased relatedness within the counties (diagonal), and pronounced relatedness between counties form squares.

We assessed the mean autosomal haplotype sharing (IBD > 3 cM) within and between counties (Fig. 2). By far the greatest within-county mean haplotype sharing was observed in Finnmark (52.2 cM), followed by Sogn og Fjordane (14.8 cM), Rogaland (14.2 cM), and Vest-Agder (13.5 cM). The marked haplotype sharing in Finnmark stands out in a Norwegian context, but elevated haplotype sharing has also been found in the Finnish population, especially eastern Finland (~45 cM) [41], suggesting homogeneity and small effective population sizes. Conversely, the smallest within-county haplotype sharing was observed for the capital area of Oslo (4.7 cM), Akershus (5.2 cM), and Østfold (5.7 cM). The greatest haplotype sharing between counties was observed for Troms and Finnmark in the North (18 cM), and for Vest-Agder and Aust-Agder in the South (10.8 cM).

**Fig. 3:**
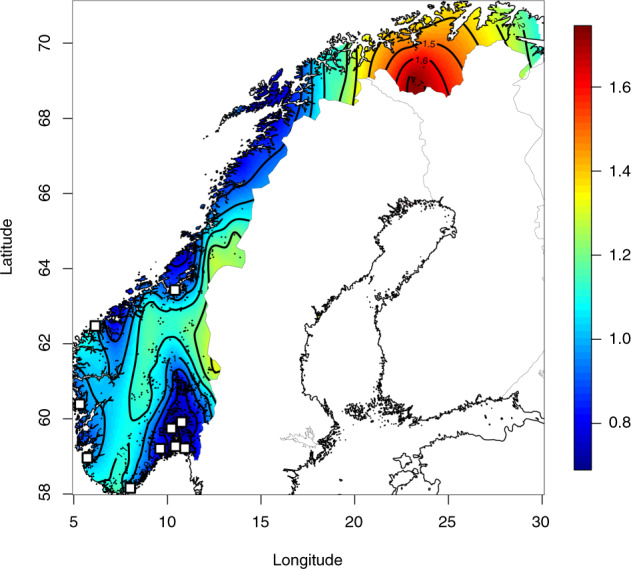
Contour plot of the cumulative sum of homozygous segments (cM) on the log10 scale detected by Beagle, extrapolated by spatial regression (Krig/fields). The black dots represent jittered coordinates of zip codes, using 2984 individuals (max 200 per county). The ten most populous cities (>50,000 inhabitants) are marked with white squares. A continuous belt of elevated homozygosity stretches along with the interior, towards the southwestern coast.

Homozygosity, measured as the summed length of homozygous segments detected by RefinedIBD, is relatively high in the north, presumably due to increased Sami and Finnish ancestry. Increased homozygosity is also evident in the border areas towards Sweden in the middle, and inland areas of mid-Norway, protruding down to the southwestern coast (Fig. 3). Areas with substantially lower degrees of homozygosity include the Oslofjord area in the southeast, the Trondheimsfjord area in the middle, and the northern county of Nordland. The county of Nordland, with no major cities and home to large fishing grounds, appears heterogeneous. We also assessed if individuals from rural areas (*n* = 1701) were significantly more homozygous than those from urban areas (20 largest cities, *n* = 1283). Individuals from rural areas were significantly more homozygous than individuals from urban areas, with a median of 6.1 cM and 5.1 cM respectively (two-sided *t*-test *p* = 9.28 × 10^–9^).

### Kinship to Denmark and Sweden

We explored the mean sum of autosomal haplotype sharing (IBD > 3 cM) between Norwegian and Swedish counties, and Denmark as a whole (Supplementary Figs. S6 and S7). We find a distinct pattern of low degree of shared ancestry between Norway and Denmark (3.1 cM), including the South/Southeast of Sweden (Skåne = 3.3 cM). At the opposite end, the northernmost county in Sweden, Norrbotten, shared 13.1 and 8.1 with Finnmark and Troms, respectively. Further, we detected elevated haplotype sharing between the counties on the border of Norway and Sweden. Noteworthy, the former disputed county of Jämtland, conquered by Sweden in 1679, stands out for having a relatively high IBD sharing with Nord-Trøndelag of 6.6 cM.

### Historical effective population sizes

The distribution of shared IBD segment lengths is also informative about *N*_e_ through time [35, 42]. Most, but not all, counties reveal a decrease in effective population sizes, with a minimum around 12–14 generations ago at 1550–1600 AD, assuming a 30-year generation time (Supplementary Fig. S4). This minimum has also been reported in other isolated populations in Northern Europe [43].

### Estimation of migrations rates

The simulations of effective migration surfaces returned numerous patterns, some of which were consistent across multiple iterations. These included a general trend of coastal pockets receiving migration and inland barriers (Supplementary Fig. S5). We observed three of the notable features. First, was an increased migration rate over a highland area entitled “Hardanger Plateau” that lies between the two largest cities in Norway, Oslo, and Bergen. This genetic corridor corresponds to known ancient trade trails and horse tracks across this highland. Second, there is evidence for barriers in the south, in line with the north-south facing valleys, coinciding with current county borders. Third, we note the isolation of the traditional Sami area of “Finnmarks Plateau” in the far north. See Supplementary Fig. S5 for a map of elevation level and locations.

## Discussion

We describe for the first time, using common variants, the genetic structure of the Norwegian population at a genome-wide scale. The Sami people, and later immigrating minorities from Finland, like the “Kven” and “Skogfinner” (~1500 AD), are recognized ethnic minorities, and their influence on the genetic landscape of Norway is clearly detectable in the PCA, especially in the three northernmost counties (Fig. 1 and Supplementary Fig. S1a). This is consistent with evidence from a health survey conducted in the 1980s in Finnmark, where ~25% of the participants reported a Finnish family background. To fully appreciate the extent of Finnish and Sami ancestry, we quantified the extent of East-Asian ancestry per county (Supplementary Figs. S1a and S2). We find a substantial extent of Asian ancestry (mean ~25%, Kautokeino), a size similar to that reported [27] in a single Sami sample (~25% Nganasan) and several Sami samples from Sweden (~30% East Asian) [44]. The northernmost county of Finnmark was disputed territory between Norway, Sweden, and Russia until 1826. Finnmark is also sparsely populated (2 per km²), with a modest recruitment area for the initial cancer study, resulting in undersampling (*n* = 30). Other under-sampled counties in our study include Troms (*n* = 54), Sogn og Fjordane (*n* = 22), and Hordaland (*n* = 52). As shown in Fig. S1b, the observation of genetic drift in Finnmark is consistent at both high and low sample sizes.

Our results further support the divergence, isolation, and homogeneity in the southern counties of Norway (Rogaland, Agder, and Telemark). The isolation is exemplified by the observation that Oslo has a relatively similar trend in historical effective population size to that of the general British population, while Rogaland had a similar historical profile to the Orkney Islands [43]. Further, the counties of Rogaland and Vest-Agder display elevated levels of within-county haplotype sharing (~13–14 cM), suggesting isolation and inbreeding (Fig. 2), as well as increased homozygosity (Fig. 3) and small *N*_e_ (Table 1). This is in line with previous reports on genetic differentiation in southern Norway [10, 11]. In this study, we have used place of residence as the geographical origin of samples, and not a place of birth, as that information was not available to us. Thus, individual relocation and patterns of the recent migration within Norway may obscure geographical stratification of genetic variance somewhat and this represents a limitation of our study.

Norway has close historical ties to Denmark, as Norway became a vassal state of Denmark in 1380, lasting 443 years, until 1814. The PCA (Supplementary Fig. S3) and IBD analyses (Supplementary Fig. S6) strongly suggest that the counties in southern Norway have diverged from the rest of the Norwegian population due to isolation, rather than gene flow from Denmark or some other neighboring populations. We speculate that the isolation in the Norwegian south may be caused by several factors. (1) The region has an unusual coastline, without deep fjords, common elsewhere in Norway. Historically the fjords have played a critical part in the transportation of goods and people. The absence of fjords may have increased isolation (2) late development of infrastructure like railroad and roads in the last 100 years (3) failure to recruit economic migrants.

In a medical context, there is a need to establish national frequency-based databases for disease studies [45]. Isolated populations may have skewed allelic frequencies and loss of variations as described for the Finnish population [46]. We have taken the first step in this endeavor by documenting geographical patterns of genetic variation in the Norwegian population. Such a database should contain a relatively large amount of frequency differences (*F*_ST_ = 0.0073) between geographical regions (Rogaland (200) vs. Finnmark [30], *F*_ST_ = 0.0073, maximum local *F*_ST_ = 0.47, rs904274) within Norway. To avoid the undesirable effects of population stratification on genotype-phenotype association studies, and to increase precision, detailed geographical information of individual origin should be included.

For the first time, we document restricted gene flow in the southern part of Norway, which is contradicting a commonly held notion of Danish admixture. We next aimed to characterize the detailed population structures in the Norwegian population further using rare variants, as rare variants are more geographically clustered, due to their more recent origin.

## Supplementary information

Supplementary data
