# Supplementary material for: The genetic structure of Norway
Source: Eur J Hum Genet. 2021 May 17;29(11):1710–8. doi: 10.1038/s41431-021-00899-6 (PMC8560852; doi:10.1038/s41431-021-00899-6)
Supplement: Supplementary file 1 — Supplementary data [file 41431_2021_899_MOESM1_ESM.docx]

Supplemental Information

|  |
| --- |
| 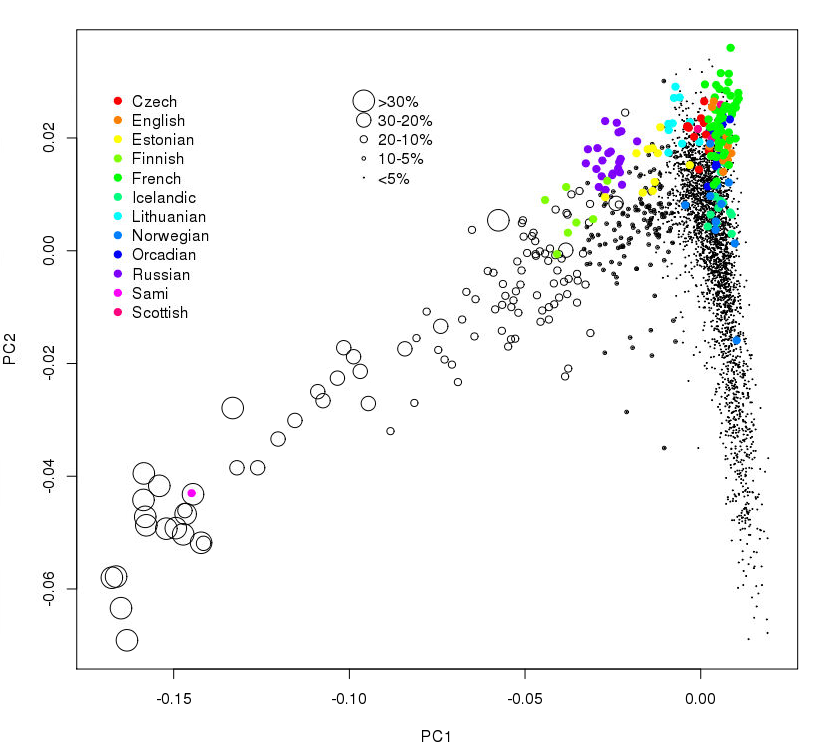  **Figure S1a:** PCA of the dataset from this study (black) merged (SNPs = 58,457) with public datasets of selected and colored European samples, including one single Sami sample from a public dataset (left legend). The size of the black circles (right legend) represents the percentage of East-Asian ancestry (CHB) calculated by ADMIXTURE. |
| 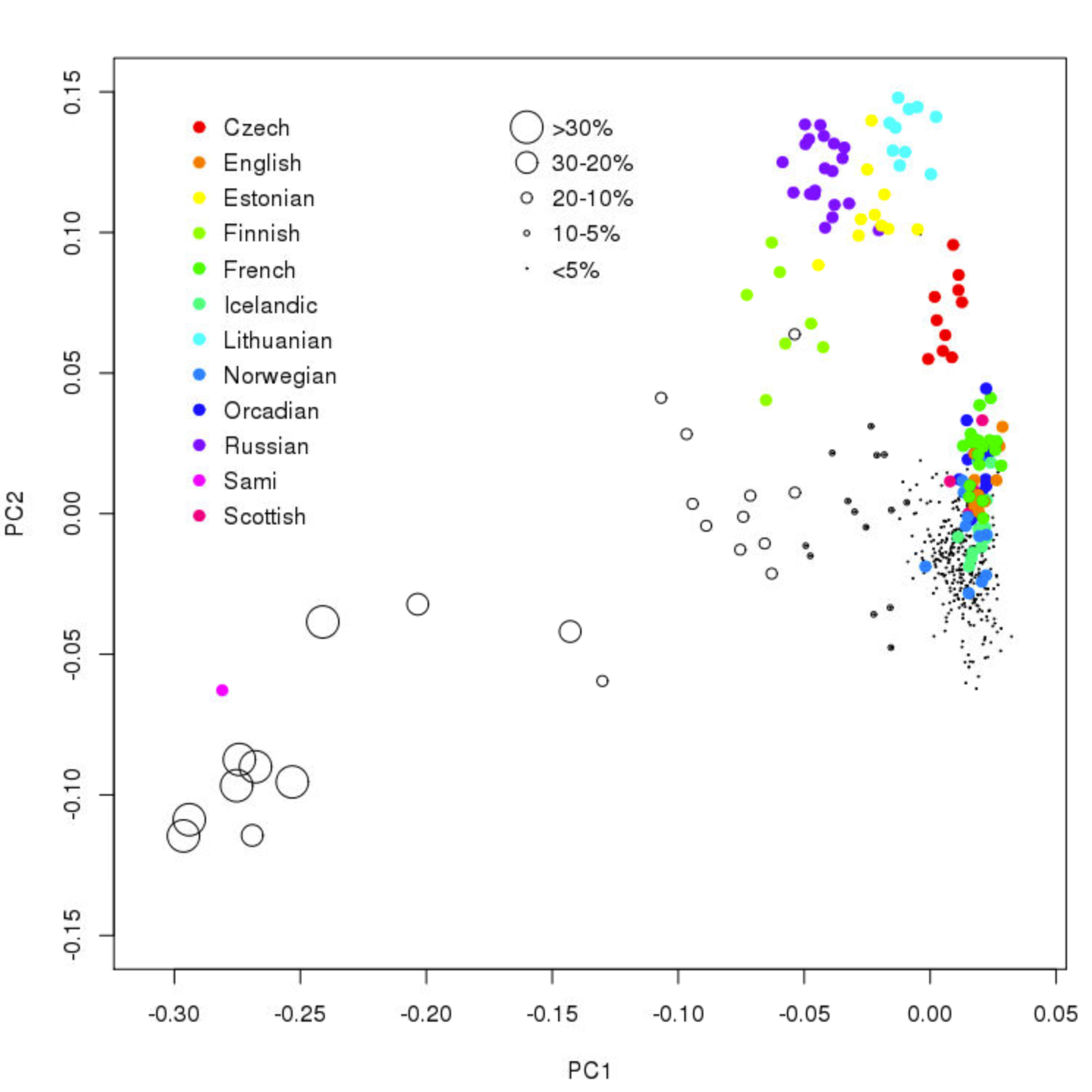 |

**Figure S1b:** Due to sample size bias, a second PCA of the dataset from this study (black) merged (SNPs = 58,457) with public datasets of selected and colored European samples. Here a maximum threshold of 20 samples per county was used, and not max. 200 as in S1a.


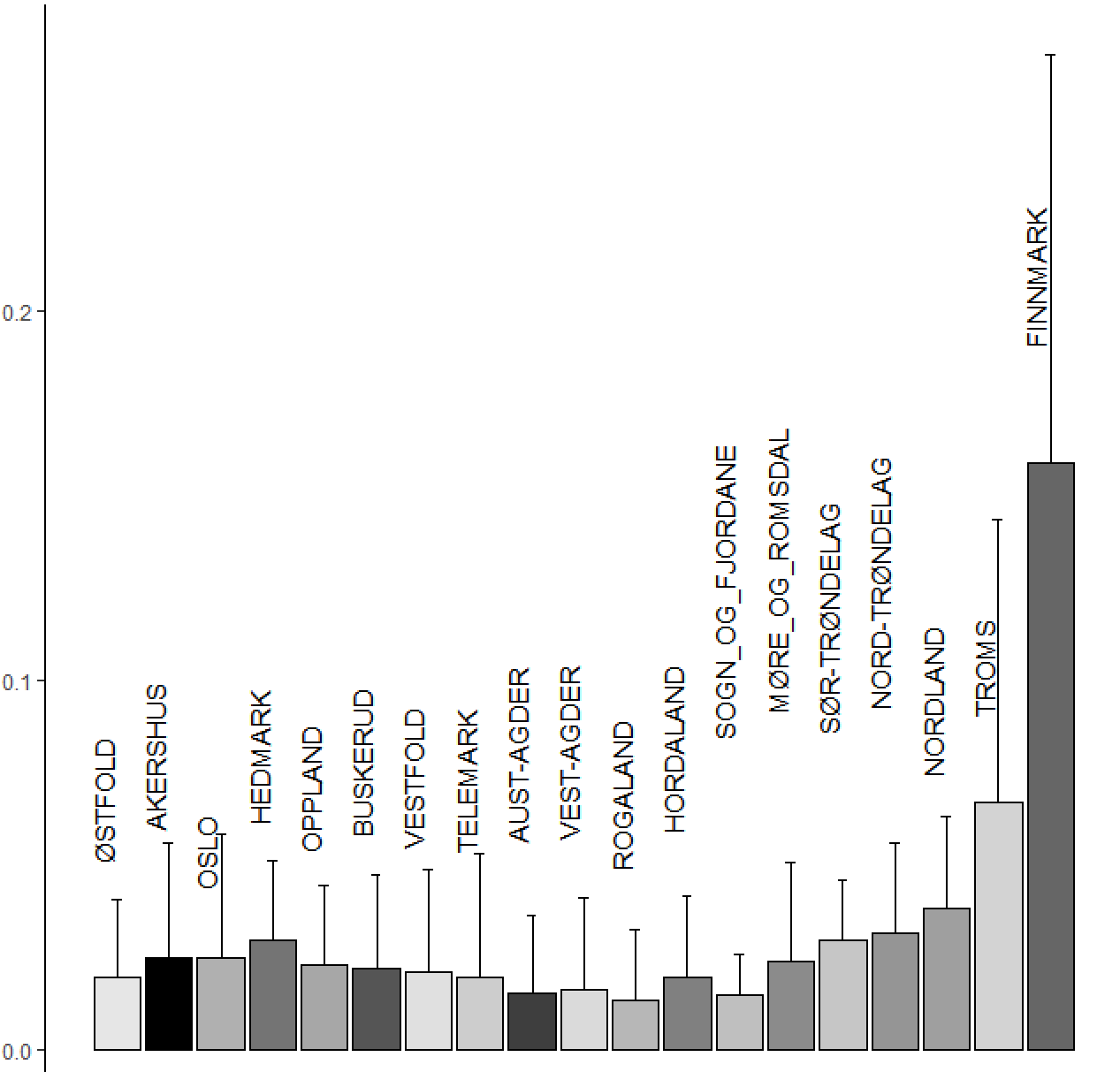


**Figure S2:** The fraction of East-Asian ancestry per county (mean with standard error of the mean) indicate increased Asian ancestry in the northmost counties of Troms and Finnmark (ADMIXTURE/ HapMap CHB).


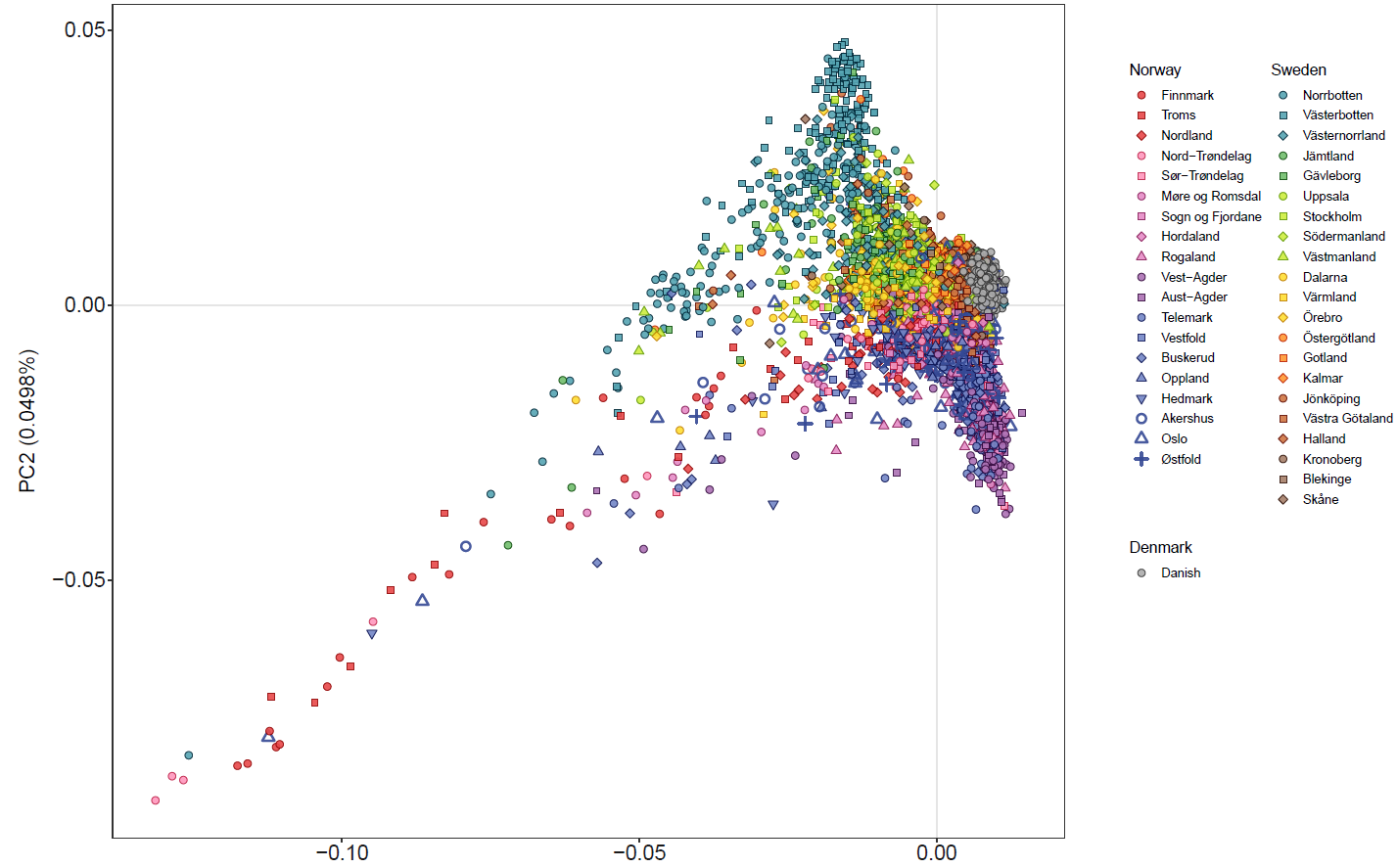


**Figure S3:** PCA plot of 8110 Scandinavian samples, consisting of 2985 Norwegians, 3519 Swedes and 1606 Danes, with regional information. A maximum of 200 samples was set per region, and LD pruned (“indep-pairwise 200 25 0.5”), leaving 238,689 SNPs. In additional to the diverging Sami/Finnish samples, samples from the northern counties of Sweden (Norrbotten and Västerbostten) and the southern counties of Norway (Rogaland, Vest-Agder, Aust-Agder and Telemark) display distinctive drift.


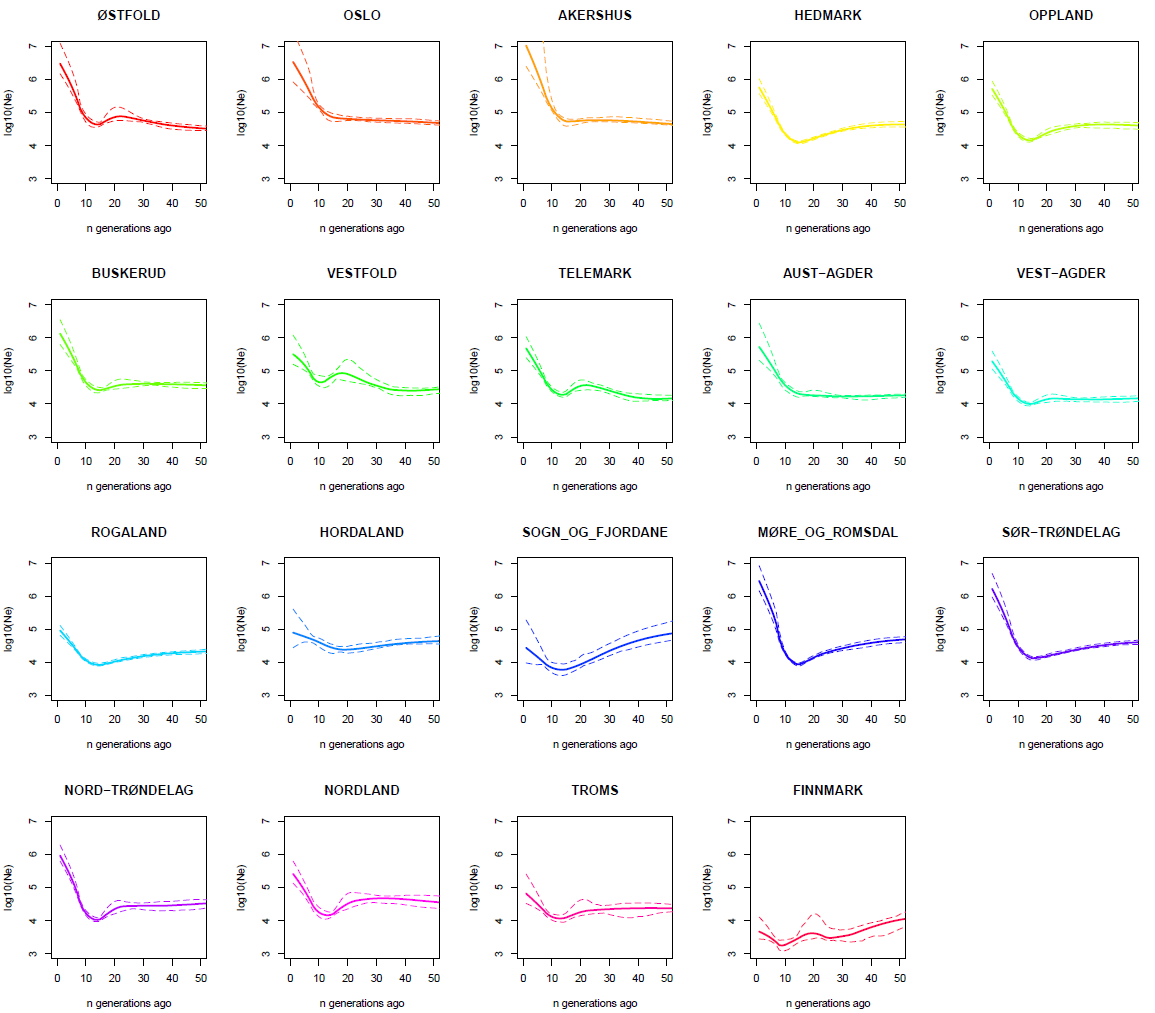


**Figure S4:** Changes in effective population sizes though time as estimated by IBDne, using IBD segments > 3 cM and maximum 50 generations back. The upper and lower 95% confidence intervals are marked with dotted lines. Most counties show a decrease in effective population sizes with a minimum around 12-14 generations ago. We assume the decline has been initiated by The Black Plague, with subsequent isolation, having a minimum at 1550-1600 AD (assuming a 30-year generation time). Counties in the far north and far south have the least growth in more recent times.

| 300 demes  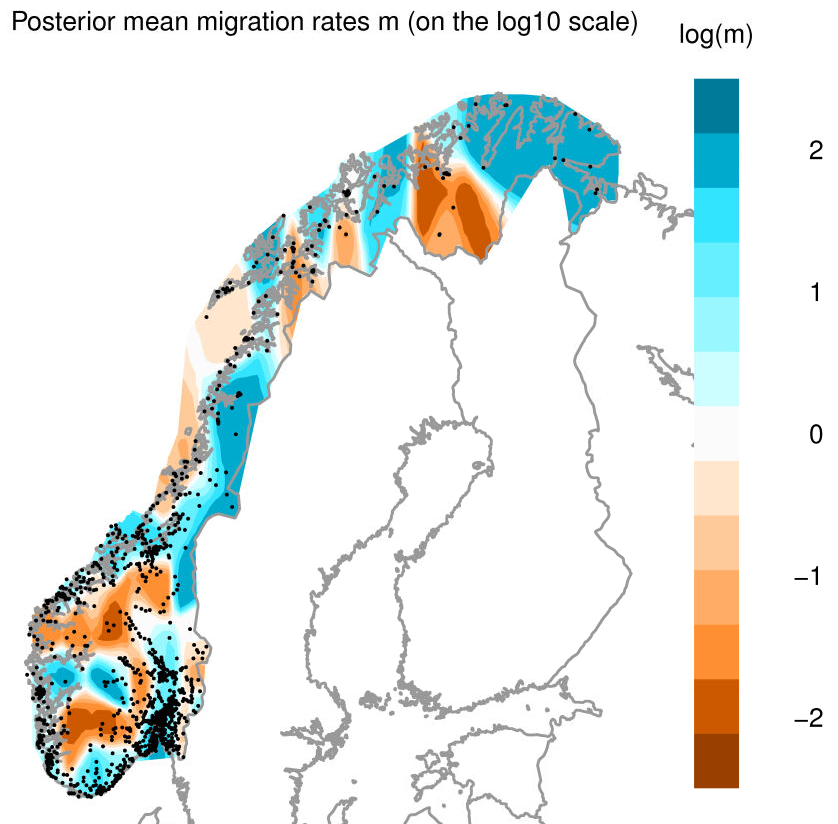 | 500 demes  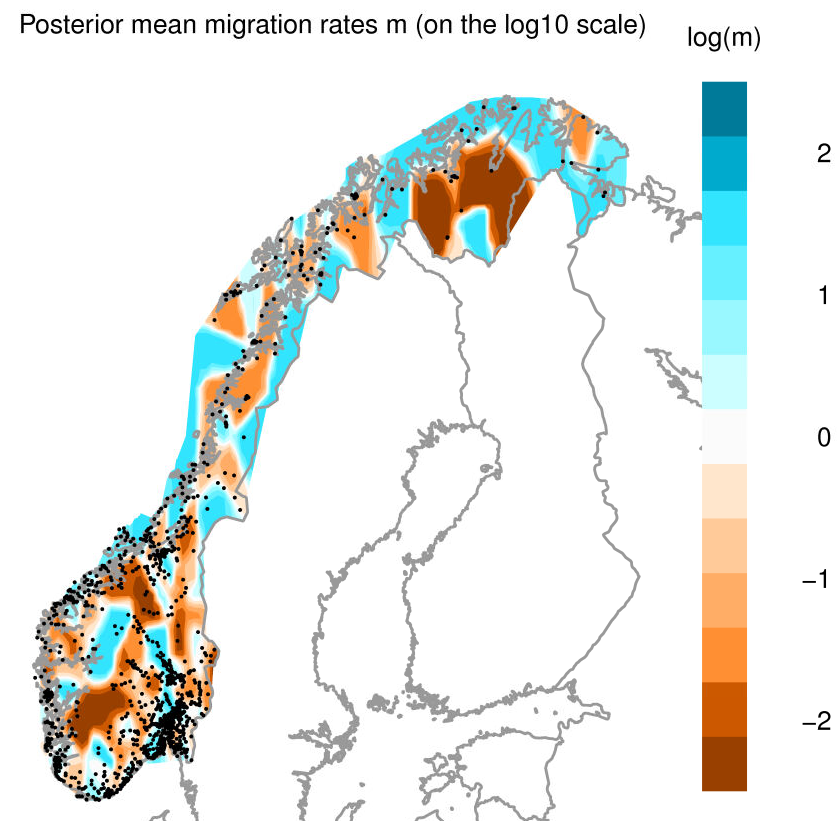 |
| --- | --- |
| 800 demes 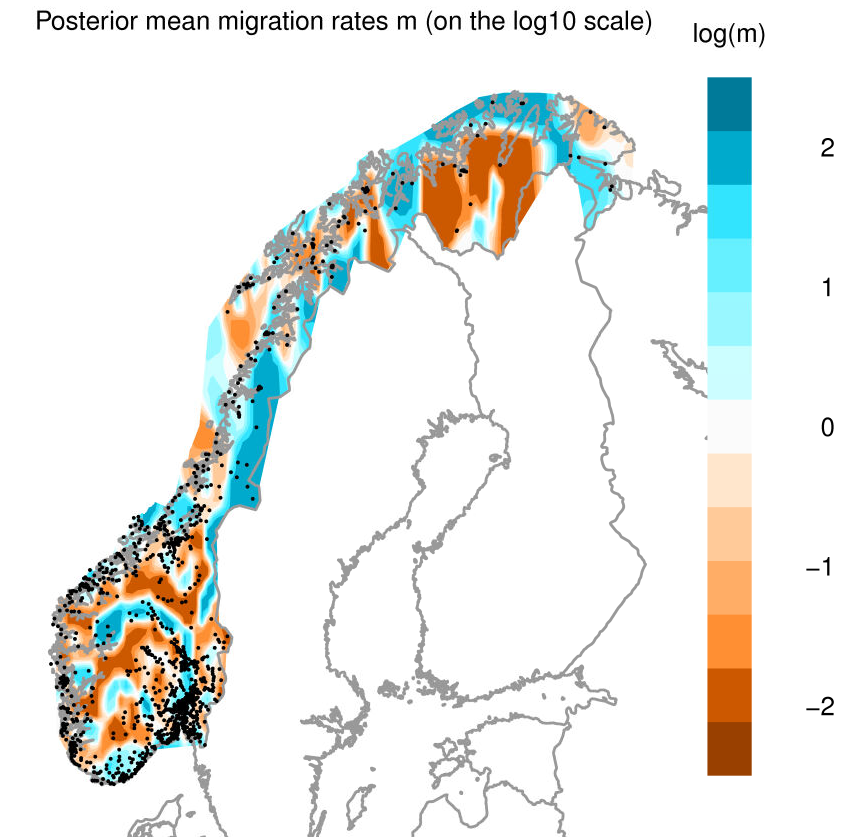 |  |

**Figure S5:** Simulation of effective migration rates using LD-pruned SNPs from 2984 (max 200 per county) individuals and 500 demes. Brown indicate areas of significantly reduced migration rates, and blue indicates significantly increased migration on a logarithmic scale. The black circles represent sample size and overlay grid.

| 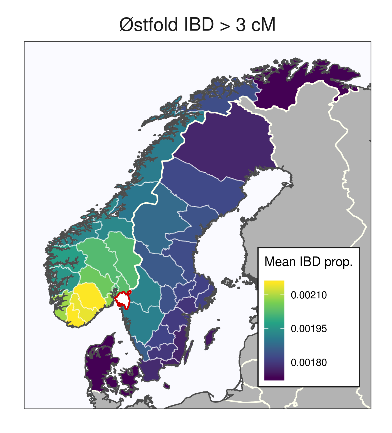 | 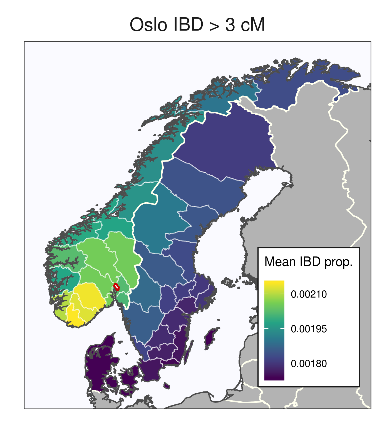 | 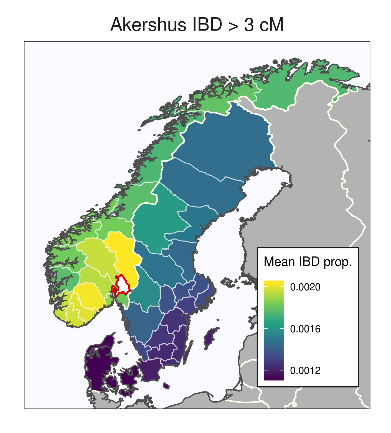 |
| --- | --- | --- |
| 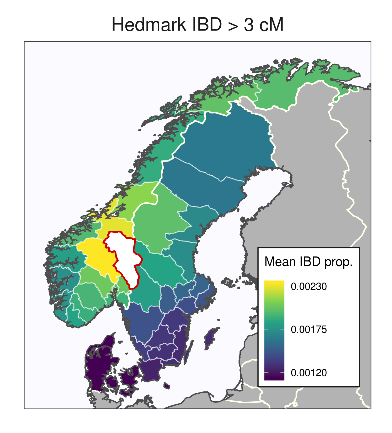 | 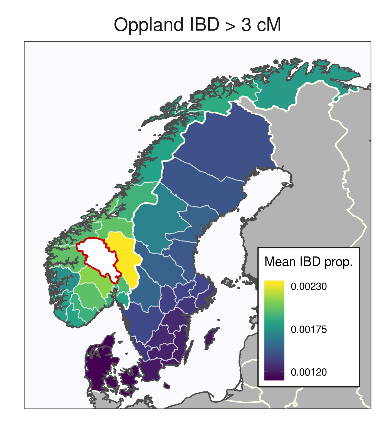 | 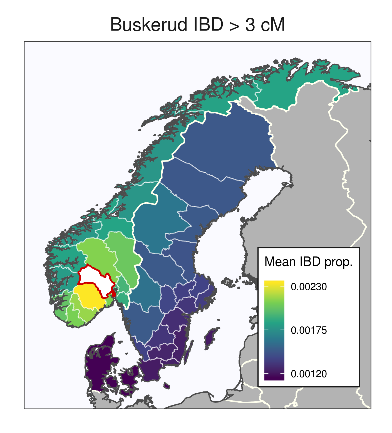 |
| 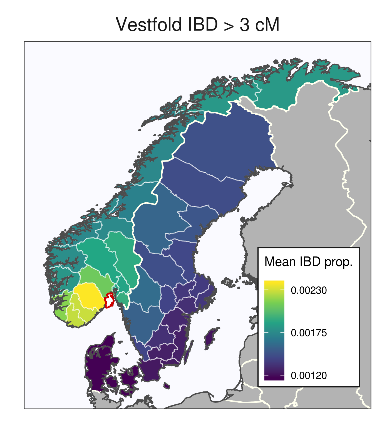 | 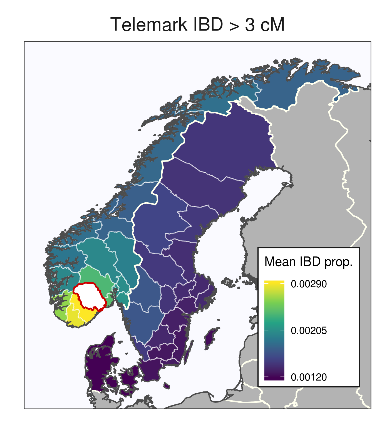 | 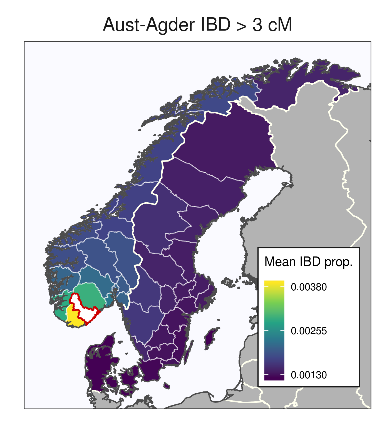 |
| 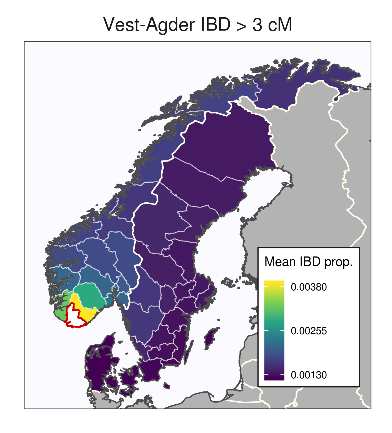 | 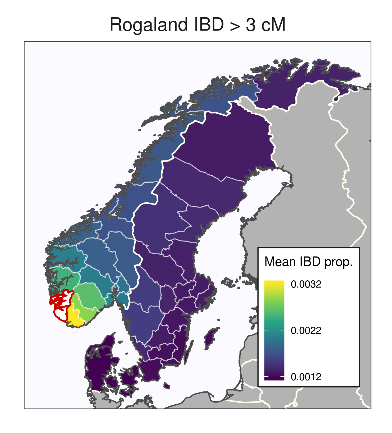 | 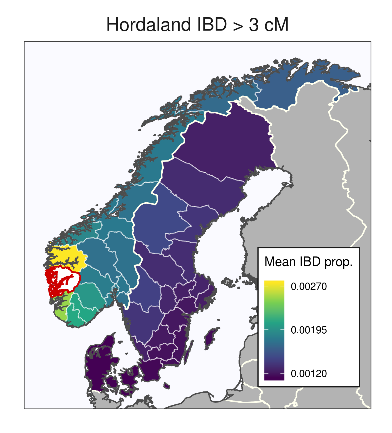 |
| 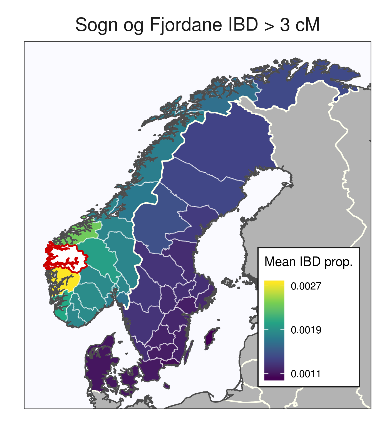 | 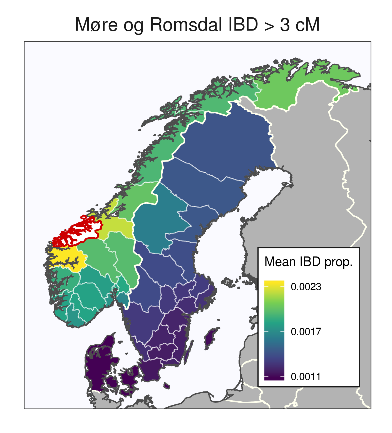 | 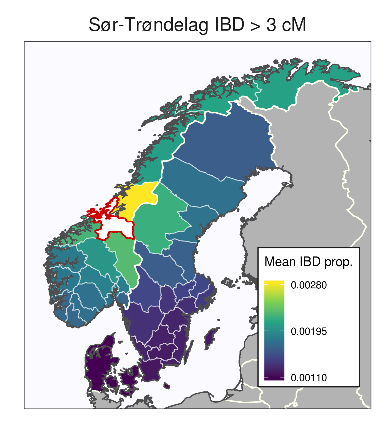 |
| 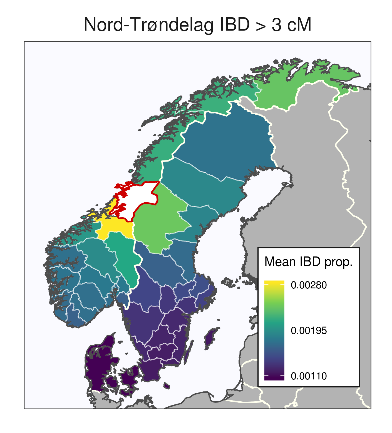 | 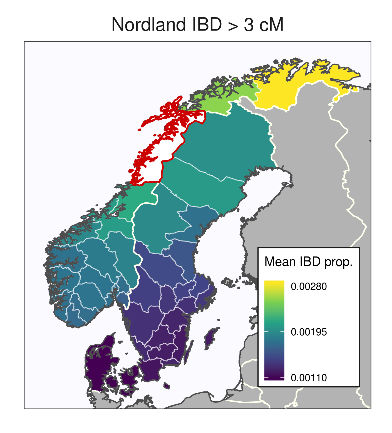 | 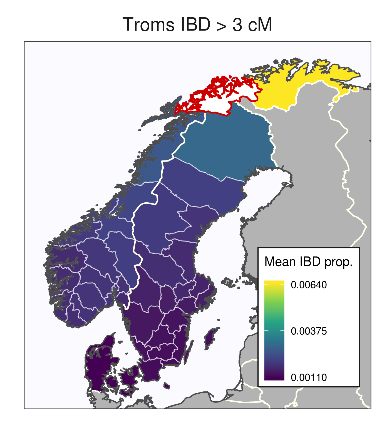 |
| 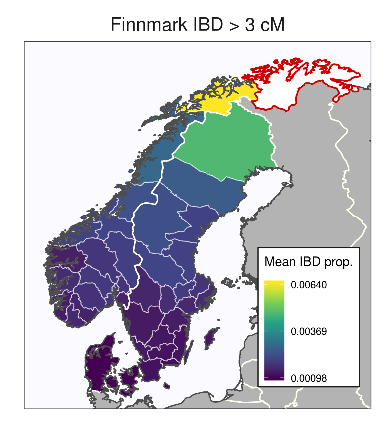 |  |  |

**Figure S6:** The proportion of shared genomic content between counties in Norway, Sweden and Denmark. The border areas between Norway and Sweden share overall more genetic content compared that of Denmark and southern Sweden.


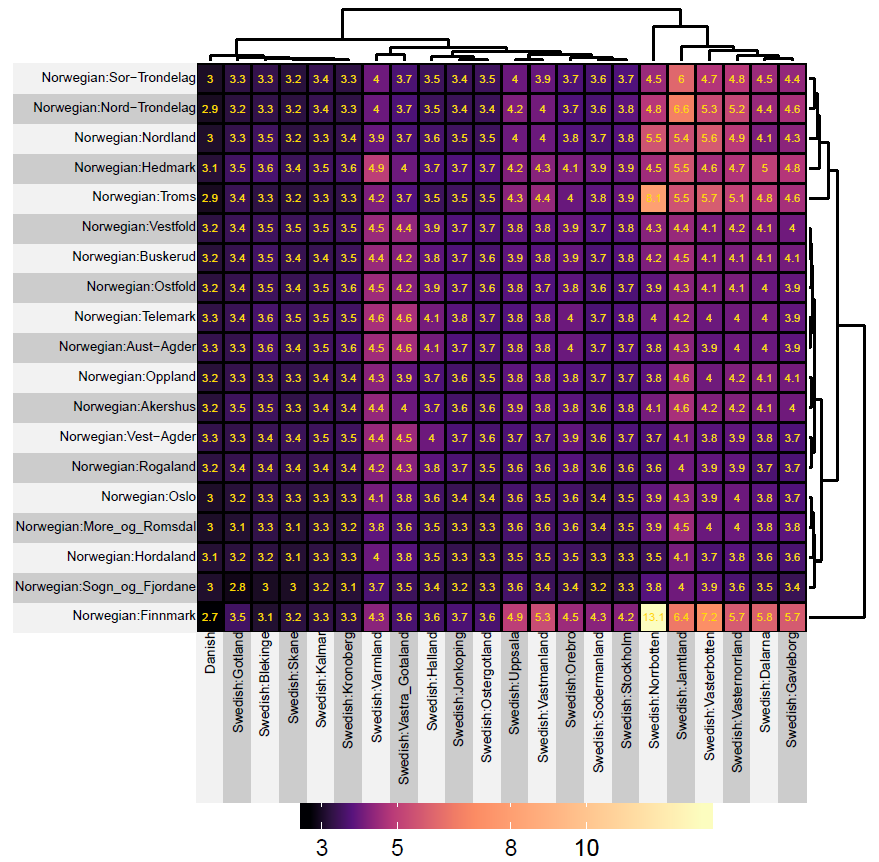


**Figure S7:** Visual representation and hierarchical clustering of the mean cumulative sum of haplotype sharing (IBD > 3cM) between counties in Norway and Sweden, including Denmark, in centiMorgans (cM). The color-coding does not scale linearly. Overall, Denmark and South/southeastern Sweden share less kinship towards Norway (dark left), than do the bordering counties between Norway and Sweden (upper right).


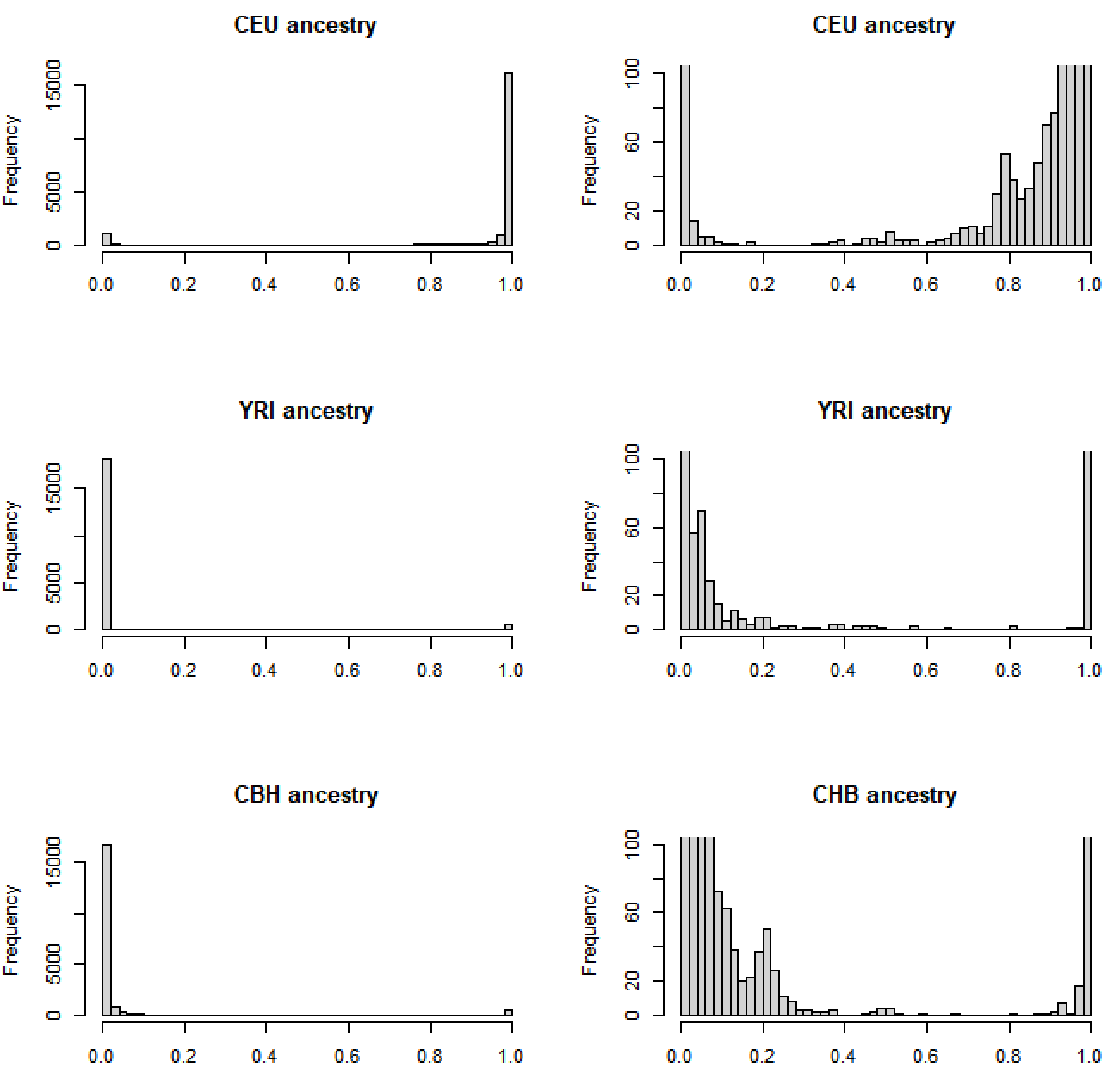


**Figure S8:** The distribution of ancestral admixture from the three HapMap populations, CEU (European), YRI (African) and CHB (Asian) used during quality control. The figures on the left show the complete distribution, while the figures of the right have truncated y axis. Note the lower right figure with an accumulation of ~0.2 CHB ancestry.


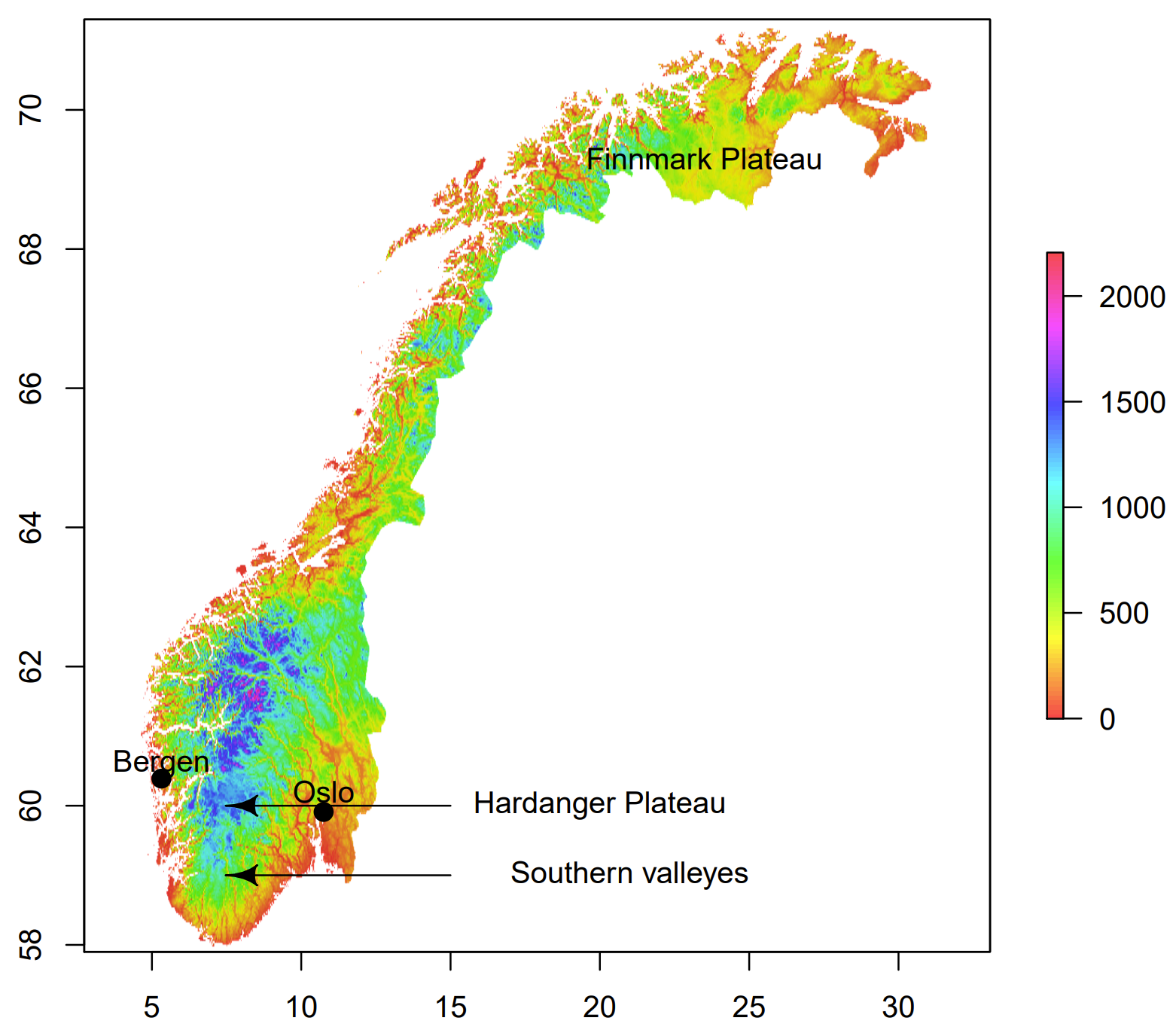


**Figure S9:** Elevation map of Norway with selected features. Broadly speaking, only red and orange areas are inhabited.


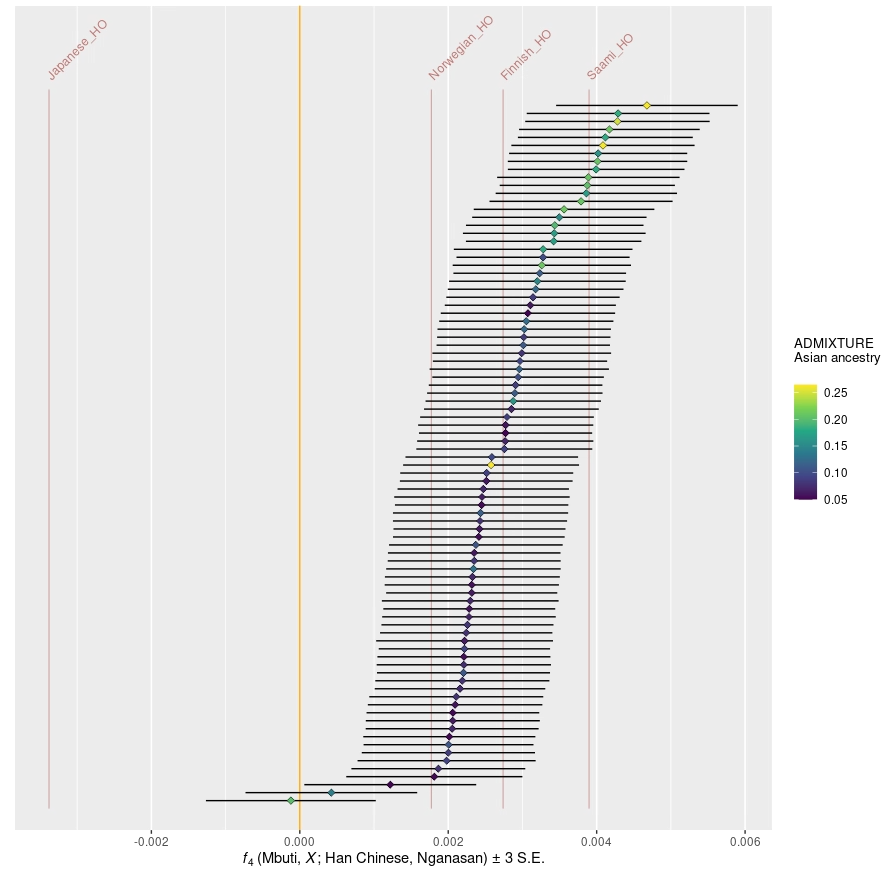


**Figure S10:** The results for the *f_4_* test on the 89 assumed Sami individuals with East Asian ancestry (5%-35%) demonstrates that the majority of assumed Sami individuals have affinity to the Nganasan and not Han Chinese. However, the ± 3 S.E. confidence interval for two individuals assigned 20 and 22% East Asian ancestry included zero, which we interpret as indicating that at least part of the East Asian ancestry in these individuals is likely non-Uralic in origin. The counties of residence for these two individuals were Akershus and Rogaland. Note the vertical red lines representing control samples from the Human Origin dataset.

Table S1

| **QC step** | **Number of samples** | **Number of SNPs** |
| --- | --- | --- |
| Starting samples number | 15,840 | 719,072 |
| SNPs with missing genotypes<2% | - | 608,817 |
| SNPs with MAF>2% | - | 596,523 |
| Samples <2% missing SNPs | 15,769 | - |
| Samples with zip code | 14,429 | - |
| Autosomal SNPs |  | 583,183 |
| Unrelated (kinship coeff.<4%, >3nd degree) | 6545 | - |
| Ancestry filter (YRI < 5%, CHB < 35%) | 6369 | - |
| Full, autosomal | 6369 | 583,183 |
| Pruned, autosomal | 6369 | 102,023 |
| Final, subset, SNP freq.-based analysis  Final, subset, haplotype analysis | 2984  2984 | 102,023  583,183 |
